# Multi-Gene Detection and Identification of Mosquito-Borne RNA Viruses Using an Oligonucleotide Microarray

**DOI:** 10.1371/journal.pntd.0002349

**Published:** 2013-08-15

**Authors:** Nathan D. Grubaugh, Scott S. McMenamy, Michael J. Turell, John S. Lee

**Affiliations:** Virology Division, United States Army Medical Research Institute of Infectious Diseases, Fort Detrick, Frederick, Maryland, United States of America; U.S. Naval Medical Research Unit No. 2, Indonesia

## Abstract

**Background:**

Arthropod-borne viruses are important emerging pathogens world-wide. Viruses transmitted by mosquitoes, such as dengue, yellow fever, and Japanese encephalitis viruses, infect hundreds of millions of people and animals each year. Global surveillance of these viruses in mosquito vectors using molecular based assays is critical for prevention and control of the associated diseases. Here, we report an oligonucleotide DNA microarray design, termed ArboChip5.1, for multi-gene detection and identification of mosquito-borne RNA viruses from the genera *Flavivirus* (family *Flaviviridae*), *Alphavirus* (*Togaviridae*), *Orthobunyavirus* (*Bunyaviridae*), and *Phlebovirus* (*Bunyaviridae*).

**Methodology/Principal Findings:**

The assay utilizes targeted PCR amplification of three genes from each virus genus for electrochemical detection on a portable, field-tested microarray platform. Fifty-two viruses propagated in cell-culture were used to evaluate the specificity of the PCR primer sets and the ArboChip5.1 microarray capture probes. The microarray detected all of the tested viruses and differentiated between many closely related viruses such as members of the dengue, Japanese encephalitis, and Semliki Forest virus clades. Laboratory infected mosquitoes were used to simulate field samples and to determine the limits of detection. Additionally, we identified dengue virus type 3, Japanese encephalitis virus, Tembusu virus, *Culex* flavivirus, and a Quang Binh-like virus from mosquitoes collected in Thailand in 2011 and 2012.

**Conclusions/Significance:**

We demonstrated that the described assay can be utilized in a comprehensive field surveillance program by the broad-range amplification and specific identification of arboviruses from infected mosquitoes. Furthermore, the microarray platform can be deployed in the field and viral RNA extraction to data analysis can occur in as little as 12 h. The information derived from the ArboChip5.1 microarray can help to establish public health priorities, detect disease outbreaks, and evaluate control programs.

## Author Summary

Approximately half of the world's population is at risk of viral, mosquito-borne illness such as dengue, yellow fever, Japanese encephalitis, Rift Valley fever, and chikungunya. In the past, these viruses have been regarded as pathogens of the tropics; however, they are emerging as global causes of illness. Very few effective drugs and vaccines have been developed for mosquito-borne viral infections and even less are available to people in resource-limited countries. An important aspect of disease prevention is mosquito surveillance to determine geographical range and seasonal prevalence of the associated viruses. However, there are hundreds of viruses transmitted by mosquitoes that are pathogenic to humans and animals. Using a portable microarray, we developed an assay with the ability to detect most of the known medically important viruses transmitted by mosquitoes. This assay was designed for use in conjunction with broad-range screening tools as a cost effective, rapid method to determine the identity of viruses from infected mosquitoes. To our knowledge, this is the most comprehensive assay to date for field surveillance of mosquito-borne viruses.

## Introduction

Arthropod-borne viruses (arboviruses) are important human and veterinary pathogens that are biologically transmitted to vertebrates by hematophagous (blood feeding) arthropod vectors, such as female mosquitoes. The diverse group of mosquito-borne RNA viruses primarily includes flaviviruses (*Flaviviridae*: *Flavivirus*), alphaviruses (*Togaviridae*: *Alphavirus*), orthobunyaviruses (*Bunyaviridae*: *Orthobunyavirus*), and phleboviruses (*Bunyaviridae*: *Phlebovirus*) [1]. Flaviviruses contain a positive-sense, single-stranded RNA genome of approximately 10.9 kilo bases (kb) in length with a single open reading frame encoding three structural and seven nonstructural proteins [2]. Mosquito-borne flaviviruses are phylogenetically divided into two groups: those transmitted by *Aedes* species mosquitoes, such as dengue virus (DENV) and yellow fever virus (YFV), and those transmitted by *Culex* species mosquitoes, such as Japanese encephalitis virus (JEV) and West Nile virus (WNV). Additionally, flaviviruses continue to be isolated from mosquitoes without a known vertebrate host, termed arthropod-specific viruses [3], [4], [5], [6], [7], [8], [9]. Alphavirus genomes are also positive-sense, single-stranded RNA molecules, approximately 11.7 kb in length that translate into four nonstructural and three structural proteins [10]. They are classified in two geographically isolated groups, Old World alphaviruses, such as chikungunya virus (CHIKV) that is found in parts of Africa, Asia, and Europe, and New World alphaviruses, such as eastern equine encephalitis and Venezuelan equine encephalitis (VEEV) viruses that circulate in the Americas. Orthobunyaviruses and phleboviruses have tripartite, single-stranded, negative-sense RNA genomes. The RNA segments, designated by their distinct sizes, small (S), medium (M), and large (L), encode for the nonstructural proteins, structural proteins, and RNA-dependent RNA polymerase, respectively [11]. Orthobunyaviruses are primarily mosquito-borne and are distributed globally. The majority of phleboviruses use phlebotomine sand flies as their primary vectors, but many can also be transmitted by mosquitoes. One notable example is Rift Valley fever virus (RVFV), which is transmitted by a number of different mosquito species in nature [12].

Arboviruses represent nearly 30% of all emerging infectious disease in the last 50 years [13]. Emergence and re-emergence of arboviral pathogens can be attributed to many factors, such as globalization, altering weather patterns, increased production of livestock, and tropical urbanization [1], [14]. The majority of emerging arboviruses are known pathogens with high epidemic and epizootic potentials when introduced into new populations, as evident by several examples: RVFV in Africa [15], [16], WNV in North America [17], [18], and CHIKV in areas near the Indian Ocean [19].With limited antiviral drugs and vaccines available, global surveillance of newly emerging and re-emerging arboviruses is critical for early detection and prevention of arboviral diseases.

An active surveillance program should include the monitoring of levels of virus activity in vector populations and in vertebrate hosts [20]. This can be challenging for a comprehensive surveillance program because mosquito-borne viruses are taxonomically diverse and expansive. Traditional polymerase chain reaction (PCR) based assays can be too limited in scope to detect unexpected circulating viruses. Microarrays, on the other hand, can detect hundreds or thousands of viral agents using oligonucleotide DNA probes [21], [22], [23]. Microarray assays have been developed to detect a wide range of viruses, including some mosquito-borne viruses [21], [22], [23], [24], [25], [26], [27], [28], [29]. These assays have been shown to be valuable tools for the detection of viral RNA in clinical samples. However, none of the assays have been designed or analyzed for use with infected mosquitoes. Most of the assays included an unbiased, random amplification method for microarray detection. We have previously reported that such techniques are efficient for the amplification viral nucleic acids isolated from cultured cells, but not from a complex matrix of RNA from mosquito homogenates [30]. Additionally, most of the microarray designs are not wide-ranging and only cover a few of the most concerning viral pathogens. Lastly, none have been evaluated for field-use.

In this report, we describe an oligonucleotide DNA microarray, the ArboChip5.1, which targets multiple genes from 144 mosquito-borne RNA viruses from the genera *Flavivirus*, *Alphavirus*, *Orthobunyavirus*, and *Phlebovirus*. For each genus, at least three sets of consensus gene-specific primers (GSPs) were designed for efficient PCR amplification of the viral nucleic acids isolated from infected mosquitoes and virus-specific microarray capture probes were designed to differentiate between the PCR amplicons. We previously demonstrated that a portable microarray platform, the ElectraSense 4×2K (CustomArray, Inc., Bothell, WA), is practical for field diagnostics [30]. The platform included a small and rugged microarray reader that was able to analyze four samples simultaneously against 2,240 oligonucleotide DNA probes using electrochemical detection (ECD) [31], [32], [33], [34]. The assay, as described here, was able to detect and identify RNA belonging to several arboviruses of medical and veterinary importance, including DENV type 3 (DENV-3), JEV, Tembusu virus (TMUV), *Culex* flavivirus (CxFV), and a Quang Binh-like virus from field-collected mosquitoes from Thailand during 2011 and 2012.

## Materials and Methods

### Ethics statement

Entomological collections from private land and residences were conducted with the owners/residents permission.

### Assay overview and method substitutions

**Figure 1:**
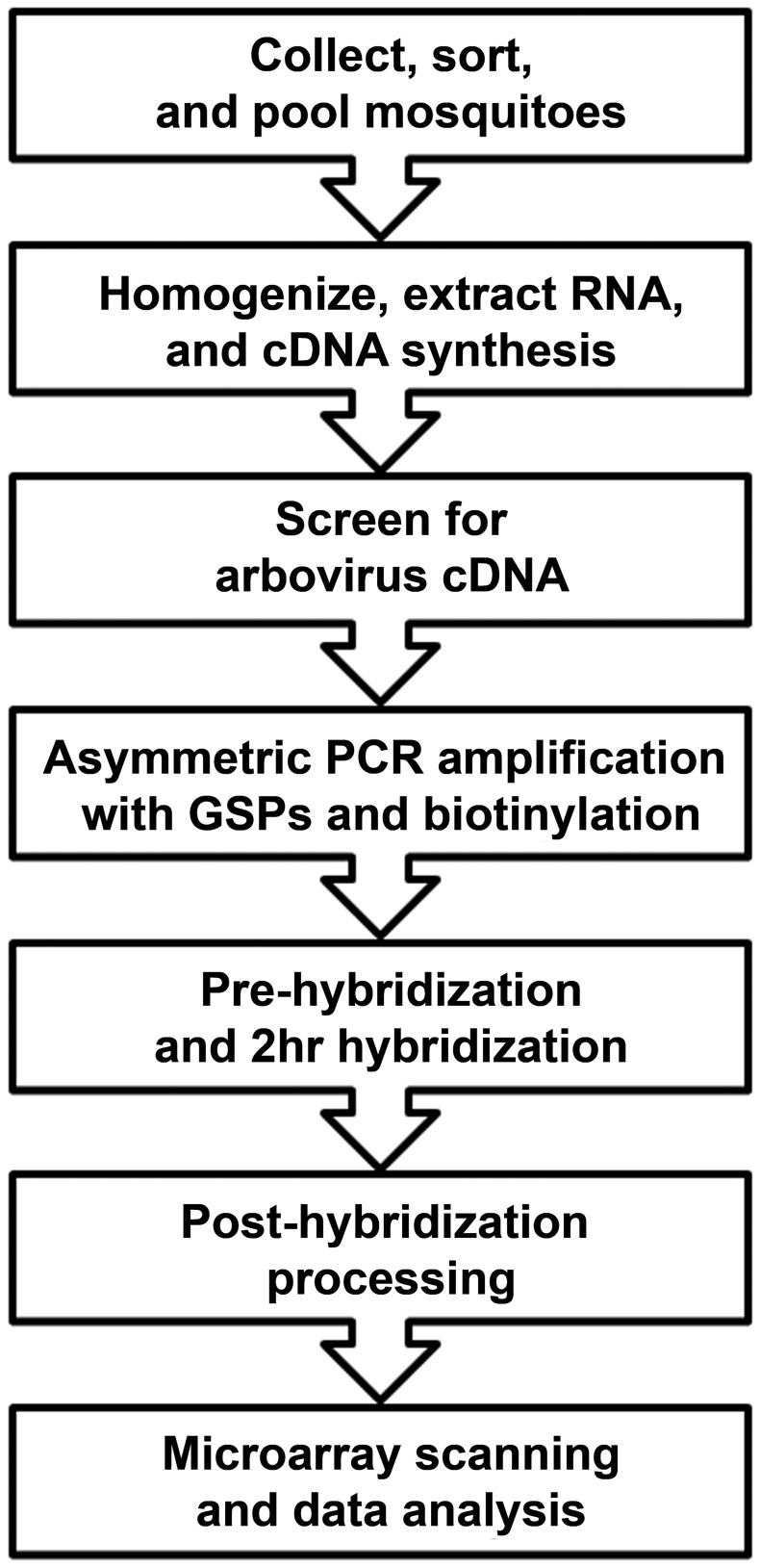
Microarray assay workflow.

The assay workflow is displayed in Figure 1. Most of the assays were completed in 12 h or less. The methods for mosquito collection, RNA extraction, and virus screening can be substituted with a group's own methods. Virus screening methods should typically include PCR assays for genus-level detection using consensus primers. Complementary DNA (cDNA) from mosquito pools that tested positive using the screening assays should continue with the assay procedures, starting at the “asymmetric PCR amplification with GSPs and biotinylation” step.

### Gene-specific primer design

Consensus GSPs for PCR amplification of microarray targets can be found in Table 1. The GSPs were modified from published assays [35], [36] or designed by aligning all microarray target sequences, according to virus genus, using the Megalign/Clustal W software (DNASTAR, Inc., Madison, WI). The GSPs were selected from conserved regions meeting the following criteria: 17–32 base pairs (bp) in length, melting temperatures between 50 and 65°C, and guanine-cytosine content (GC-content) between 45 and 65%.

**Table 1:** Gene-specific primers (GSPs) for PCR amplification of microarray targets.

Virus genus	Gene/segment	Primer name	GSP sequence (5′ to 3′)	Product (bp)	GenBank ID
*Flavivirus*	E	F1269-F	GAGGCTGGGGAAATGGCTG	969	NC_002031
		F2225-R	CCTCCAACTGATCCAAAGTCCCA		
	NS3	F5015-F	GTGGTTGGNCTGTATGGNAA	812	
		F5807-R	CCCATTTCTGAGATGTCAGT		
	NS5	F8276d-F^c^	AAYTCNACNCANGARATGTAY	804	
		F9063d-R^c^	CCNARCCACATRWACCA		
*Alphavirus*	nsP1	A183d-F^d^	TCCATGCHMAYGCBAGRGCDTTYTCGCA	937	NC_004162
		A1095d-R	GTNGCHADDATNCCNGTCATYTGRT		
	nsP4	A6493d-F	CCRYTDCANSAVRTACCNATGGA	1009	
		A7482d-R	CHATYYAGGWYMRCCGTASA		
	E1	A10240d-F	GGBGTNTACCCNTTYATGTGGGG	914	
		A11135d-R	ATGTGGTCYKYHGGDGGNT		
*Phlebovirus*	S	PR-S1192d-F	TCAATRAKRCCAGCAAAGCTRGGATGCATC	391	NC_002045
		PR-S1558d-R	GGGTSMAWGAGTTTGCTTAYCAAGG		
	M	P-M2292d-F	TGYAGRGARGGNMMKAGYTTYTGGAC	689	NC_002044
		P-M2955d-R	CCATTCCTVRCYTGWGGYARAGANCC		
	L	P-L1992d-F	GGARGAYAARGCWRYMACWGAAG	1068	NC_002043
		P-L3039d-R	GGAWGDGTGAAYTCRCAYARC		
*Orthobunyavirus*	S BUNV^a^	OB-S121-F	CACCAGCAGTACTTTTGACCCAGA	649	NC_001927
		OB-S746d-R	TTTAGCHARGAABTCYCTRGCWGC		
	S CEV^b^	OC-S127d-F	TTTRAYCCNGAKGCAGGGTWTGTGG	484	U12797
		OC-S594d-R	GCYTTCTTCAGGWACTKDGSRTCCATC		
	M	O-M3266d-F	GGNTGTGTWTWTGGNTCWTGYCARGA	906	NC_001926
		O-M4147d-R	CCTTCATCYYKNACYCTRCAYADCC		
	L	O-L3156-F	GCAGATATGTCAAAATGGAGTGCTC	1088	NC_001925
		O-L4223-R	CTCATGTCACTTGTTTCACCC		

The primer start position, based on the GenBank source, is listed in the primer name. The “d” in the primer name designates that degenerate nucleotides were included. “F” and “R” at the end of the primer name designates forward and reverse, respectively. BUNV, Bunyamwera virus; CEV, California encephalitis virus.

a S segment primers for BUNV and Wyeomyia clade targets.

b S segment primers for CEV, Bwamba, and Simbu clade targets.

c Previously published as Flav100F and Flav200R [35].

d Modified version of VIR966-F [36].

### Microarray capture probe design

Probes were designed to target unique viral sequences, 30 to 45 nucleotides in length, between the GSPs using methods previously described [30]. A total of 2,097 oligonucleotide probes were selected for inclusion on the ArboChip5.1. The probe set included: 802 targeting flaviviruses, 307 targeting alphaviruses, 572 targeting orthobunyaviruses, and 381 targeting phleboviruses. Positive hybridization (n = 10) and negative background control (n = 25) probes were added to the design as previously reported [37]. Multiple copies of the control probes were added to fill the 2,240 probe sites present on the sectored microarray chip. Table S1 displays all the targeted viruses and the number of probes for each virus selected for inclusion on the ArboChip5.1. The complete list of capture probe sequences can be found in Table S2. All of the probes included on the ArboChip5.1 design were evaluated with earlier design versions and found to not hybridize with unintended targets, i.e., cDNA from uninfected cultured cells, uninfected mosquitoes, and viruses from different genera (unpublished data). Due to a lack of sequence information at the time of probe design, probes for all three gene targets were not included for some viruses. Probes targeting viruses not tested in this publication should be considered investigational until evaluated. The oligonucleotide probes were synthesized directly on the ElectraSense 4×2K sectored microarray by CustomArray, Inc.

### Microarray probe grouping

The virus-targeted capture probes were sorted into 12 groups, three groups for each genus based on the gene target. Each group was further sorted into subgroups based on the virus clade and probe specificity. For example, a probe specific for the DENV-3 NS5 gene would be in the *Flavivirus* NS5 group and the DENV clade, DENV-3 subgroup (DENV_DENV3). Probes that hybridized to multiple, but related viruses were classified as non-virus-specific and sorted into genus (i.e. flavivirus_generic) or clade-specific (i.e. DENV_clade) subgroups. Some very closely related viruses, such as O'nyong-nyong virus (ONNV) and Igbo Ora virus (IOV) of the Semliki Forest virus (SFV) clade, could not be differentiated. Probes for virus targets that could not be differentiated were sorted into subgroups containing virus complexes (i.e. SFV_ONNV/IOV).

### Laboratory viruses and mosquito infections

The laboratory viruses used in this study are listed in Tables 2–5. Viruses were propagated primarily in Vero (African green monkey kidney) or C6/36 (*Aedes albopictus*) cell cultures. Mosquitoes were inoculated intrathoracically (0.3 µL/mosquito) with selected viruses at approximately 10^5^ plaque forming units (PFU)/mL [38]. Inoculated mosquitoes were held for at least 7 days at 26°C to allow for virus replication. Virus-inoculated mosquitoes were triturated individually in diluent (Eagle's minimal essential medium containing 10% heat-inactivated fetal bovine serum, 0.075% NaHCO_3_, and 100 units of penicillin and 100 µg of streptomycin per mL). All virus preparations were tested in duplicate using the microarray assay.

**Table 2:** Microarray reproducibility.

Virus	Run 1	Run 2	Run 3	Run 4	Mean	SD	95% CI	CV
West Nile L1	14.29	14.95	14.91	14.89	14.76	0.27	14.32, 15.19	1.8%
Chikungunya	16.59	19.84	21.11	23.23	20.19	2.41	16.36, 24.02	11.9%
Rift Valley fever	26.78	25.53	29.96	29.54	27.95	1.85	25.0, 30.9	6.6%

Microarray analysis using cell-cultured derived viruses were performed in four replicates. The z-scores of the virus-specific probes were averaged and listed for each run. The mean and standard deviation (SD) was calculated to determine the 95% confidence interval (CI) and coefficient of variance (CV) for each virus.

**Table 3:** PCR amplification and microarray detection of flaviviruses.

		PCR amplification/microarray detection^a^			
Virus	Strain	E	NS3	NS5	Mosquito species^b^
Bussuquara	RV270	POS^d^/yes	POS^c^/yes	POS^c^/yes	n.t.
Chaoyang	ROK144	POS/yes^d^ ^e^	POS/yes	POS/yes	n.t.
Dengue type 1	HAW	POS/yes	POS/yes	POS/yes	*Ae. aegypti*
Dengue type 2	S16803	POS/yes	POS/yes	POS/yes	*Ae. aegypti*
Dengue type 3	Thai 1987	POS/yes	POS/yes	POS/yes	*Ae. aegypti*
Dengue type 4	CAR 341750	POS/yes	POS/yes	POS/yes	*Ae. albopictus*
JE	Th9-0175	POS/no	POS/yes	POS/yes	*Cx. pipiens*
Kunjin	R4336a	NEG	POS/yes	POS/yes	n.t.
MVE	RV241	POS/yes	POS/yes	POS/yes	n.t.
Quang Binh	Th5-0215	POS/no	POS/yes	POS/yes	n.t.
Rocio	SP H34675	NEG	POS^c^/yes	POS^c^/yes	n.t.
SLE	Ft. Washington	POS^c^/yes	POS/yes	POS/yes	n.t.
Tembusu	Th3-0385	POS/yes	POS/yes	POS/yes	*Cx. tarsalis*
Tembusu	Th6-0381	POS/yes	POS/yes	POS/yes	n.t.
West Nile L1	EG101	POS/yes	POS/yes	POS/yes	n.t.
West Nile L1	NY397-99	POS/yes	POS/yes	POS/yes	*Cx. pipiens*
West Nile L2	KLF 76	POS/yes	POS/yes	POS/yes	n.t.
West Nile L2	KLF 146	POS/yes	POS/yes	POS/yes	n.t.
Yellow fever	17D	POS^c^/yes	POS/yes	POS/yes	*Ae. aegypti*
Zika	30306	NEG	POS/yes	POS/yes	n.t.

Viral RNA derived from cell culture or infected mosquitoes were PCR amplified using GSPs. Amplicons were analyzed using the ArboChip5.1 microarray. JE, Japanese encephalitis; MVE, Murray Valley encephalitis; SLE, St. Louis encephalitis; L1, lineage 1; L2, lineage 2; POS, positive PCR amplification; NEG, negative PCR amplification; yes, detected by microarray; no, not detected by microarray; n.t., not tested.

a Viruses propagated in cell culture and identified by microarray to species unless otherwise noted.

b Virus-infected mosquito used for microarray evaluations, microarray detected at least one target for each virus.

c PCR amplification produced a weak visible band.

d ArboChip5.1 does not include probes specific to the target.

e Microarray detection with genus-level probes only.

**Table 4:** PCR amplification and microarray detection of alphaviruses.

		PCR amplification/microarray detection^a^			
Virus	Strain	nsP1	nsP4	E1	Mosquito species^b^
Aura	RIID 1990	POS/yes	POS/yes	POS/yes	n.t.
Babanki	Ken07-46A-49	POS/yes^f^	POS/yes^f^	POS/yes^f^	n.t.
Chikungunya	INDO23574	POS/yes	POS/yes	POS/yes	*Ae. taeniorhynchus*
Getah	ROK-2.0017	POS/yes	POS/yes	POS^c^/yes	n.t.
Mayaro	TR467	POS/yes	POS/yes	POS/yes	n.t.
Ndumu	Ken07-332	POS/yes^d^ ^e^	POS/yes	NEG^d^	n.t.
Ockelbo	ISL-44	POS/yes^f^	POS/yes^f^	POS/yes^f^	n.t.
O'nyong nyong	Gulu	POS/yes^g^	POS/yes^g^	POS/yes^g^	n.t.
Ross River	T-49	POS^c^/yes	POS^c^/yes	NEG	*Ae. albopictus*
Semliki Fortest	Ken07-586	POS/yes	POS/yes	POS/yes	n.t.
Sindbis	Ken07-611	POS/yes^f^	POS/yes^f^	POS/yes^f^	*Ae. aegypti*
Una	PE-1.0800	POS/no^d^	POS/yes	POS/no^d^	n.t.
VEE	TC83	POS/yes	POS/yes	POS/yes	n.t.
WEE	McMillan	POS/yes	POS/yes	POS/no	*Ae. albopictus*

Viral RNA derived from cell culture or infected mosquitoes were PCR amplified using GSPs. Amplicons were analyzed using the ArboChip5.1 microarray. VEE, Venezuelan equine encephalitis; WEE, western equine encephalomyelitis; POS, positive PCR amplification; NEG, negative PCR amplification; yes, detected by microarray; no, not detected by microarray; n.t., not tested.

a Viruses propagated in cell culture and identified by microarray to species unless otherwise noted.

b Virus-infected mosquito used for microarray evaluations, microarray detected at least one target for each virus.

c PCR amplification produced a weak visible band.

d ArboChip5.1 does not include probes specific to the target.

e Microarray detection with genus-level probes only.

f Target was detected but could not be differentiated between BBKV, OCKV, and SINV.

g Target was detected but could not be differentiated between ONNV and IOV.

**Table 5:** PCR amplification and microarray detection of phleboviruses.

		PCR amplification/microarray detection^a^			
Virus	Strain	S	M	L	Mosquito species^b^
Rift Valley fever	ZH501	POS/yes	POS/yes	POSe/yes	*Cx. pipiens*
Rift Valley fever	ZH548	POS/yes	POS/yes	POSe/yes	n.t.
Sandfly fever Naples	85-055	POS/yes	POS/no^c^	POS/yes^c^ ^d^	n.t.
Toscana	ISS PHI3	POS/yes	POS/yes	POS/yes	n.t.
Candiru	BeH2251	POS/yes	POS/yes	POS/yes	n.t.
Chagres^e^	LW10	POS/no^c^	POS/no^c^	POS/yes^c^ ^d^	n.t.

Viral RNA derived from cell culture or infected mosquitoes were PCR amplified using GSPs. Amplicons were analyzed using the ArboChip5.1 microarray. POS, positive PCR amplification; NEG, negative PCR amplification; yes, detected by microarray; no, not detected by microarray; n.t., not tested.

a Viruses propagated in cell culture and identified by microarray to species unless otherwise noted.

b Virus-infected mosquito used for microarray evaluations, microarray detected at least one target for each virus.

c ArboChip5.1 does not include probes specific to the target.

d Microarray detection with genus-level probes only.

e Not a target virus on ArboChip5.1.

### Isolation of RNA and synthesis of cDNA

TRIzol-LS (Invitrogen Inc., Carlsbad, CA) extraction of RNA and cDNA synthesis using random hexamers and SuperscriptII (Invitrogen Inc.) was completed as previously described, except that the RNA was not subjected to a second round of purification before cDNA synthesis [39].

### Asymmetric PCR amplification and biotin labelling of microarray targets

The sequences of the GSPs are listed in Table 1. Each sample was amplified using the appropriate virus genus GSP set as determined by virus screening. Asymmetric PCR amplification and biotin labelling of the microarray targets was accomplished as follows. For a 25 µL reaction, 2 µL of cDNA, 1 µL of forward primer (10 µM), 5 µL of reverse primer (10 µM), 3 µL of biotin-14-dCTP (0.4 mM) (Invitrogen Inc.), and 14 µL of nuclease-free water was added to each PCR tube containing one puRe Taq Ready-To-Go™ PCR bead (Amersham Biosciences, Corp., Piscataway, NJ). The thermocycling conditions were set as follows: 95°C for two min; 8 cycles of: 94°C for 15 sec, 56–42°C (starting at 56°C, reducing 2°C each cycle) for 30 sec, and 72°C for 60 sec; 32 cycles of: 94°C for 15 sec, 40°C for 30 sec, and 72°C for 60 sec; followed by 72°C for 7 min and a final hold at 4°C. Amplicons were visualized using 2% Agarose E-Gel gels that contained ethidium bromide (Invitrogen Inc.), as previously described [39]. Expected product sizes are listed in Table 1.

### Preparation of positive controls for hybridization

Ten microarray probes (Table S2) and reverse compliment oligonucleotides were used as positive controls for hybridization. The 10 positive oligonucleotide controls (100 µM) were combined equally resulting in a final concentration of 10 µM for each oligonucleotide. Ten microliters of the positive control pool was biotin labelled using the Label IT® μArray Biotin Labelling Kit (Mirus Bio LLC, Madison, WI) following the vendor's instructions. The biotin-labelled positive control pool was purified using the MinElute PCR Purification kit (QIAGEN Inc., Valencia, CA) following the manufacturer's instructions and eluted from the purification columns using 20 µL of nuclease-free water and 1 µL was used to spike each sample before hybridization (see below).

### Microarray hybridization and electrochemical detection

The ArboChip5.1 microarray chips were hydrated using phosphate-buffered saline (PBS) (pH 7.4) for 10 min at 65°C and then pre-hybridized for 5 min at 50°C in pre-hybridization buffer (6× SSPE [0.9 M NaCl/60 mM sodium phosphate/6 mM EDTA], 0.05% Tween-20, 14 mM EDTA, 5× Denhardt's solution, 0.05% sodium dodecyl sulfate (SDS)) with rotation using a UVP HB-500 Minidizer hybridization incubator (Ultra-Violet Products, LLC, Upland, CA) that was modified with microarray clamps fixed onto the rotisserie wheel. Preparation of the DNA samples for microarray hybridization was completed as follows. In a PCR tube, 15 µL of asymmetric biotin-labelled PCR amplicons (5 µL of each of the three gene PCR amplicons, including products without a visible band of the expected size) and 1 µL of biotin-labelled positive hybridization control oligonucleotides were mixed with 15 µL of 2× hybridization buffer (12× SSPE, 0.1% Tween-20, 28 mM EDTA, 0.1% SDS). The samples were denatured for 1 min at 95°C, cooled for 1 min at 4°C, and then added to the microarray chambers. The microarray samples were hybridized at 50°C for 2 h with rotation. The microarray chambers were rinsed with 2× PBST (2× phosphate-buffered saline pH 7.4, 0.1% Tween-20), re-incubated with the same solution at 50°C for 5 min with rotation, and washed two more times with 2× PBST. The chambers were blocked with ElectraSense Blocking Buffer for 15 min at room temperature (RT) and labelled with ElectraSense Biotin Labeling Solution for 15 min at RT. The chambers were washed twice with ElectraSense Biotin Wash Solution, incubated for 10 min at RT, washed a third time with the Biotin Wash Solution, and rinsed with ElectraSense TMB Rinse Solution. The samples were developed using ElectraSense TMB Substrate and the ECD signals were measured in picoamps using the ElectraSense Reader within 1 min of adding the substrate. Data were recorded using the ElectraSense application software. All ElectraSense products were purchased from CustomArray, Inc. and the hybridization and detection methods were based on their recommendations, except that the 16 h hybridization was reduced to 2 h.

### Microarray data analysis

The data were transformed into text files and transferred to Microsoft Excel (Microsoft Corp., Redmond, WA) for analysis. Each capture probe was sorted into one group, consisting of the virus genus and gene or segment, and one subgroup, consisting of the virus clade and specific target. The virus clades were based on observed phylogenetic analysis and did not always correspond to other reported clades or serogroups. The specific targets were single viruses or groups of related viruses.

The ECD signals were transformed into standard scores (z-scores) by subtracting the average signal of the negative background controls from each measured probe and dividing the difference by the standard deviation of the negative controls. Aforementioned, the probes were sorted into groups and subgroups for analysis. Subgroups with average z-scores greater than 10 were considered positive and used for viral RNA identification. Z-scores greater than 10 indicate that the measured probe values were greater than 10 standard deviations above the background, therefore significant. Graphs for each group expressing the subgroups' average z-scores, maximum individual probe z-scores, and the positive cut-off (10) were created for visual analysis. Example data analysis is shown in Dataset S1.

### Microarray stripping

In order for the microarray chips to be re-hybridized with new PCR amplicons, the previous amplicon:probe hybrids were denatured and then the amplicons were washed off. This was accomplished by washing the chip with Stripping Solution (CustomArray, Inc.), incubating the chip in the same solution at 65°C for 1 h, and then washing the chips with 95% EtOH and nuclease-free water. The microarray chambers were filled with PBS until re-use. The microarray chips were not used more than five times.

### Determination of the lower limits of detection and comparison to real-time and conventional PCR

The microarray lower limit of detection (LLOD) for virus infected mosquitoes was evaluated by using 10-fold serial dilutions of RNA extracted from one infected mosquito pooled with 24 uninfected mosquitoes. Isolation of RNA and synthesis of cDNA was completed using the methods described. The mosquito pool dilutions were tested using the described microarray amplification and detection methods and compared to results obtained using corresponding real-time PCR (qPCR) and convectional PCR assays. Real-time PCR was completed as follows: a 20-µL reaction contained 10 µL of SYBR Premix *Ex Taq* DNA Polymerase (Takara Bio Inc., Otsu, Japan), 0.4 µL of 10 µM forward primer, 0.4 µL of 10 µM reverse primer, 7.2 µL of nuclease-free water, and 2 µL of cDNA template using the following primer sets: mFU1 and cFD2 primers for the detection of flaviviruses [40], VIR2052 primers for the detection of alphaviruses [36], and RVS and RVAs primers for the detection of RVFV [41]. The cycling conditions were as follows: 95°C for 30 sec, then 40 cycles of 95°C for 5 sec and 60°C for 20 sec. Fluorescence was read at the end of the 60°C annealing-extension step. Conventional PCR was completed as previously described [39] using the following broad-range primer sets: MA and cFD2 primers (260 bp amplicon) for the detection of flaviviruses [42], VIR2052 primers (120 bp amplicon) for the detection of alphaviruses [36], and Phlebo forward 1, forward 2, and reverse primers (370 bp amplicons) for of the detection of phleboviruses [43].

### Mosquito field-collections and virus screening

Mosquitoes were collected from locations near Lopburi and Kamphaeng Phet, Thailand, during March and April of 2011 and near Kamphaeng Phet and Ranyong, Thailand, during April and May of 2012. The mosquitoes were identified, processed, tested for the presence of viral RNA using the broad-range conventional PCR assays for flaviviruses, alphaviruses, and phleboviruses described above. In addition, BSC82V and BSC332V primers (250 bp amplicons) were used to screen for the Bunyamwera virus (BUNV) group [44]. The ABI 3100 genetic analyzer and Big Dye 3.1 (PE Biosystems, Inc., Foster City, CA) was used to sequence the amplicons.

## Results

### Assay specificity and reproducibility

The GSP sets did not PCR amplify viruses from a different genus, uninfected cells (C6/36, BHK, and Vero cells), or uninfected mosquitoes (using species listed in Tables 3–6). The negative PCR reactions were still tested on the microarray and none were detected. Fifty-two virus strains from 46 different species were evaluated for PCR amplification and probe specificity, representing 46/144 (32%) of the total targeted viruses. Each microarray probe was evaluated for specificity to its intended target(s). Some PCR amplicons cross-hybridized to non-predicted probes (i.e. probes specific for a different virus or group of viruses); however, in each case there was no more than one such probe per subgroup and the average z-scores were never greater than 10. No probes cross-hybridized with viruses from a different genus.

**Table 6:** PCR amplification and microarray detection of orthobunyaviruses.

		PCR amplification/microarray detection^a^				
Virus	Strain	S BUNV	S CEV	M	L	Mosquito species^b^
Bunyamwera	330	POS/no^c^	NEG^d^	POS/yes	POS/yes	n.t.
Bunyamwera	131B-06	POS/no^c^	NEG^d^	POS/yes	POS/yes	n.t.
Bunyamwera	460	POS/no^c^	NEG^d^	POS/yes	POS/yes	n.t.
Bwamba	6502	NEG^e^	POSd/yes	NEG^c^	NEG^c^	n.t.
Cache Valley	RV257	POS/yes	NEG^d^	POS/yes	POS/no^c^	n.t.
Germiston	SAAr1050	POS/no	NEG^d^	POS/yes	POS/no^c^	n.t.
Ilesha	Zika-54	NEG	NEG^d^	POS/yes	NEG^c^	n.t.
La Crosse	97-WV-131	NEG^d^	POS/yes^c^ ^e^	POS/yes	POS/yes	*Ae. aegypti*
Maguari	MSP-18	NEG^c^	NEG^d^	NEG	POS/no^c^	n.t.
Oropouche	TR9760	NEG^d^	POS/yes	NEG	POS/yes	n.t.
Pongola	SA Tar	NEG^d^	POS/yes	NEG^c^	NEG^c^	n.t.
Tahyna	RV285	NEG^d^	POS/yes	POS/no	POS/yes	n.t.

Viral RNA derived from cell culture or infected mosquitoes were PCR amplified using GSPs. Amplicons were analyzed using the ArboChip5.1 microarray. POS, positive PCR amplification; NEG, negative PCR amplification; yes, detected by microarray; no, not detected by microarray; n.t., not tested.

a Viruses propagated in cell culture and identified by microarray to species unless otherwise noted.

b Virus-infected mosquito used for microarray evaluations, microarray detected at least one target for each virus.

c ArboChip5.1 does not include probes specific to the target.

d GSP set not specific for this clade.

e CEV clade detection only.

The measured variability between replicates of the same samples was used to determine if the assay results were reproducible. Cell-culture derived WNV lineage 1 (WNVL1, strain NY397-99), CHIKV (strain INDO23574), and RVFV (strain ZH501) were PCR amplified using the GSP sets and analyzed by the microarray four times independently. The z-scores from the virus-specific probes were averaged for each replicate. The mean, standard deviation, 95% confidence interval and coefficient of variance was calculated for each virus. The results are summarized in Table 2.

### Amplification, detection, and identification of flaviviruses

**Figure 2:**
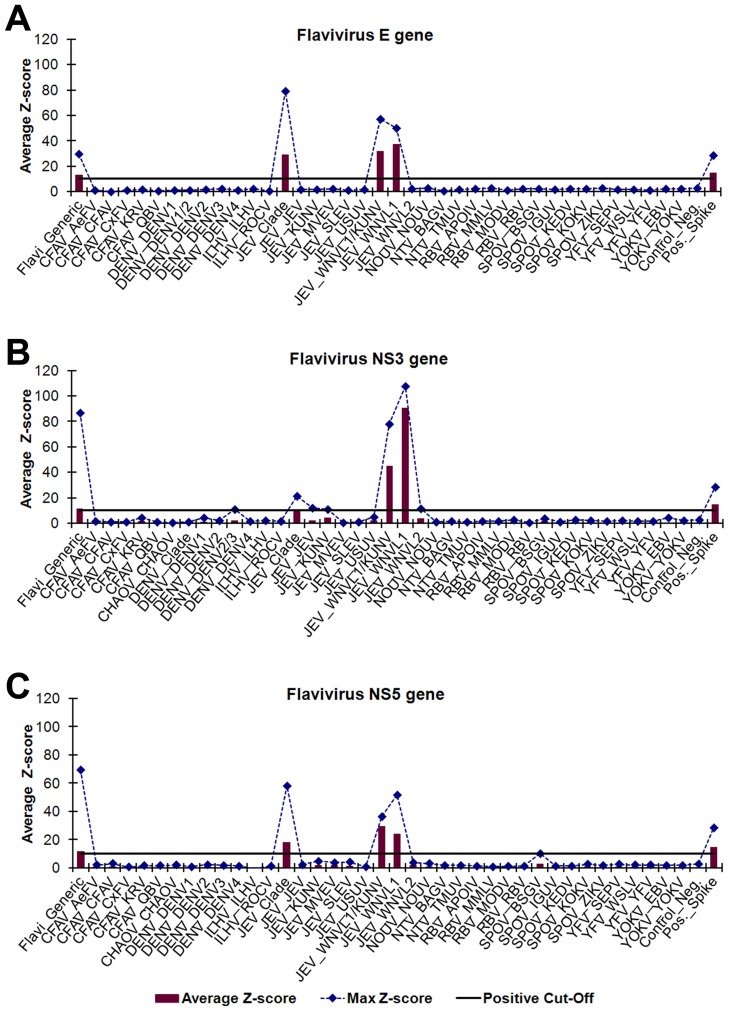
Visual analysis of cell-culture derived WNVL1 strain NY397-99. *Flavivirus* gene-specific PCR amplicons were identified using oligonucleotide microarray probes grouped by virus genus and gene, (A) *Flavivirus* E gene, (B) *Flavivirus* NS3 gene, and (C) *Flavivirus* NS5 gene, and sorted into subgroups based on phylogenetic clade and target virus(es). ECD signals were converted into z-scores. Subgroups with average z-scores greater than 10 were considered positive and used for virus identification. The plotted maximum z-scores represent the greatest individual probe z-score within a subgroup and were used to determine cross-hybridization. The PCR amplicons hybridized with probes in the *Flavivirus* generic, JEV clade, WNVL1/KUNV, and WNVL1 subgroups for all three gene targets. WNVL1 was differentiated from KUNV by the less than 10 z-scores for the KUNV subgroups. The virus abbreviations are defined in Table S1.

Microarray identification of PCR amplicons using the consensus flavivirus GSPs was evaluated using 20 flaviviruses propagated in cell culture (Table 3). A visual analysis example of WNVL1 (strain NY397-99) is shown in Figure 2. All of the flaviviruses tested were identified to species via the NS3 and NS5 gene targets and 15/20 flaviviruses (75%) were detected by all three gene targets. The Kunjin virus (KUNV), Rocio virus (ROCV), and Zika virus (ZIKV) strains tested did not PCR amplify using the flavivirus E gene GSPs. The JEV and Quang Binh virus (QBV) strains tested did amplify using the flavivirus E gene GSPs but were not detected by the microarray E gene probes. Multiple strains of TMUV, WNVL1, and WNVL2 were used to evaluate microarray identification of virus targets containing slight nucleotide differences. The 17 different flaviviruses evaluated represents 17/34 (50%) of the total flaviviruses targeted by the ArboChip5.1.

Single mosquitoes infected with DENV types 1–4, TMUV, JEV, WNVL1, and YFV were evaluated (virus strains and mosquito species are listed in Table 3). Overall, the results were similar to those obtained using the viruses propagated in cell culture, with the following exceptions. The PCR bands for DENV-4 infected *Ae. albopictus* were faint but they were still accurately identified by all three genes on the microarray. The E gene was not PCR amplified from either stain of TMUV infected *Culex tarsalis* and could not be detected on the microarray. To mimic field samples, single mosquitoes infected with DENV-1, TMUV, and WNVL1 were pooled with 24 negative mosquitoes. Compared to single infected mosquitoes, the identification results were the same but had overall lower z-scores. A WNVL1-infected and a JEV-infected *Cx. pipiens* were pooled with 23 negative mosquitoes to assess the amplification and detection of two distinct viruses that could be found in a field collected pool of mosquitoes. All three gene targets were amplified using the flavivirus GSP sets but the E genes of JEV and WNVL1 and the NS5 gene of WNVL1 were not detected using the microarray. Three of the five JEV-specific NS5 gene probes had z-scores greater than 10, but the average z-score for the subgroup was less than 10 (9.13). However, all of the JEV- and WNVL1-specific (out of 5 and 8, respectively) and two of the four WNVL1/KUNV NS3 probes had z-scores greater than 10 (Figure S1). The JEV-specific probes and the WNVL1-specific probes did not hybridize to NS3 amplicons from the other virus when evaluated individually; therefore this is an example of microarray detecting the RNA from two related but distinct flaviviruses in the same mosquito pool.

### Amplification, detection, and identification of alphaviruses

**Figure 3:**
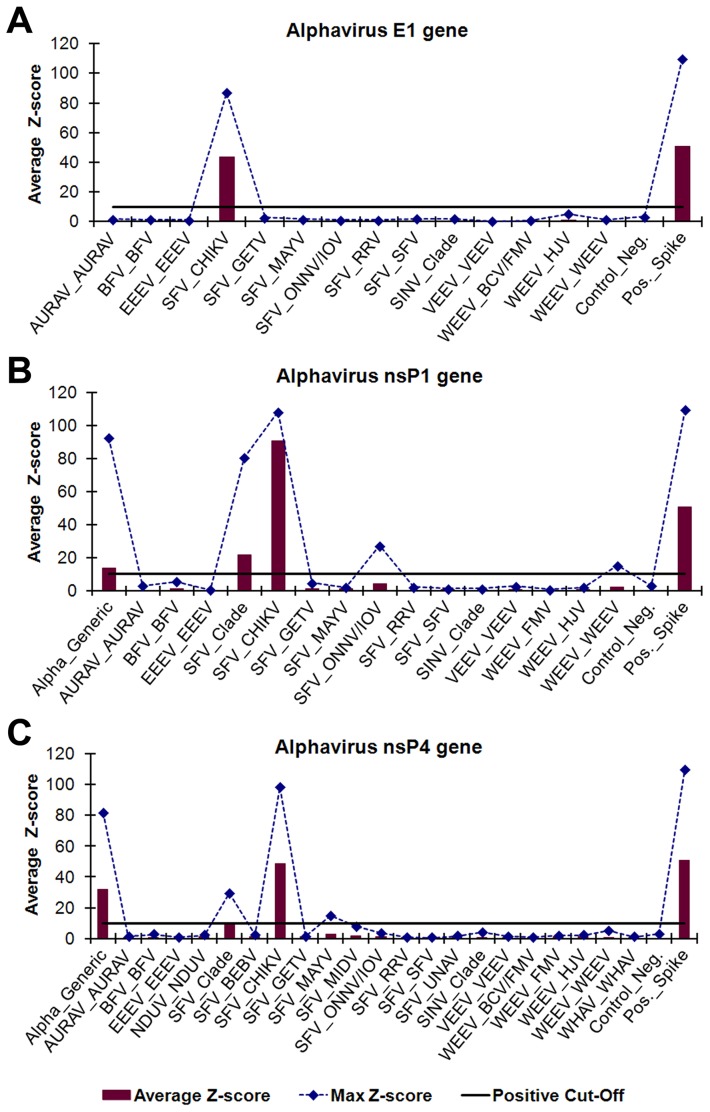
Visual analysis of cell-culture derived CHIKV strain INDO23574. *Alphavirus* gene-specific PCR amplicons were identified using oligonucleotide microarray probes grouped by virus genus and gene, (A) *Alphavirus* E1 gene, (B) *Alphavirus* nsP1 gene, and (C) *Alphavirus* nsP4 gene, and sorted into subgroups based on phylogenetic clade and target virus(es). ECD signals were converted into z-scores. Subgroups with average z-scores greater than 10 were considered positive and used for virus identification. The plotted maximum z-scores represent the greatest individual probe z-score within a subgroup and were used to determine cross-hybridization. The PCR amplicons hybridized with probes in the CHIKV subgroups for all three gene targets and probes in the *Alphavirus* generic and SFV clade subgroups for the nsP1 and nsP4 genes. The virus abbreviations are defined in Table S1.

Microarray identification of PCR amplicons using the consensus alphavirus GSPs was evaluated using 14 alphaviruses propagated in cell culture (Table 4). A visual analysis example of CHIKV (strain INDO23574) is shown in Figure 3. All of the alphaviruses were identified to species via detection by the nsP1 and nsP4 gene targets and 10/14 (71%) were identified by all three gene targets. However, Ndumu virus (NDUV) and Ross River virus (RRV) were not PCR amplified using the alphavirus E1 GSP set and the Una virus (UNAV) nsP1 and E1 amplicons were not detected by the microarray probes. Subspecies of Sindbis virus (SINV), Ockelbo virus (OCKV) and Babanki virus (BBKV), could not be differentiated from SINV and the probes for all three viruses were placed in the “SINV_clade” subgroup. Likewise, ONNV and IOV, a subspecies of ONNV, could not be differentiated and the probes were placed into the same subgroup. The 14 alphaviruses evaluated represents 14/23 (61%) of the total alphaviruses targeted on the microarray design.

To mimic field samples, *Aedes* species mosquitoes infected with CHIKV, RRV, SINV, and western equine encephalomyelitis virus (WEEV) were evaluated individually and pooled with 24 uninfected mosquitoes (virus strains and mosquito species are listed in Table 4). As expected, the z-scores were the greatest when using PCR amplicons generated from cell culture propagated virus and were approximately twofold greater compared to using PCR amplicons from individual infected mosquitoes. Single infected mosquitoes pooled with 24 uninfected mosquitoes produced the lowest z-scores; however, the z-scores were still greater than 10 resulting in positive virus identification.

### Amplification, detection, and identification of phleboviruses

**Figure 4:**
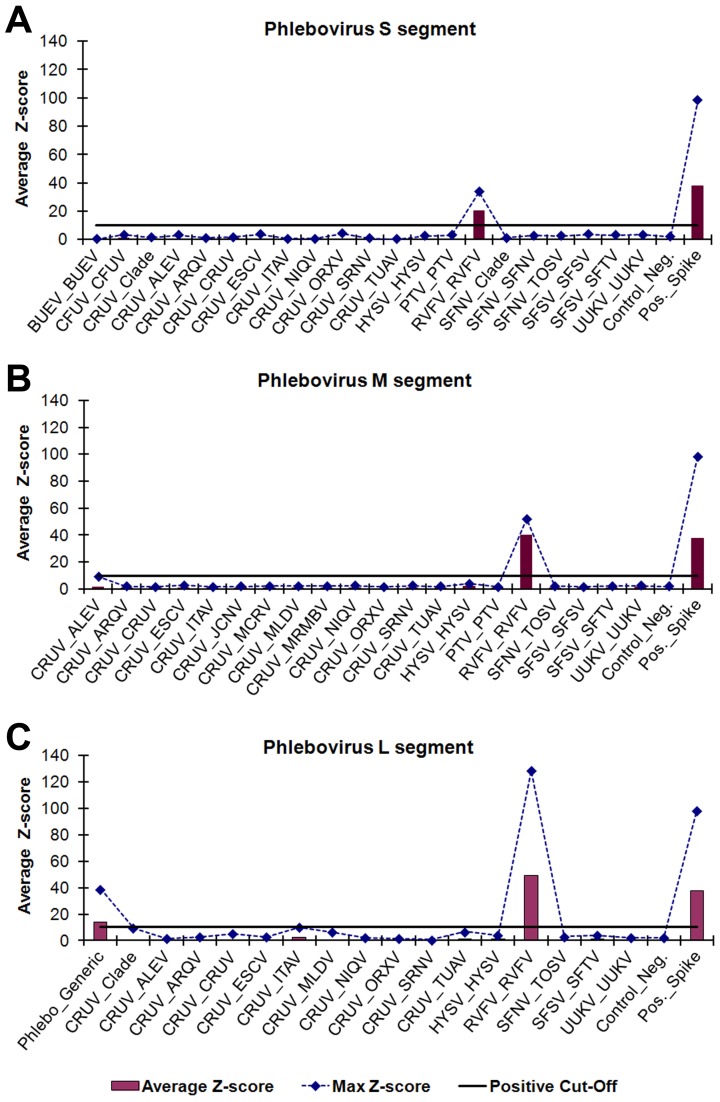
Visual analysis of cell-culture derived RVFV strain ZH548. *Phlebovirus* gene-specific PCR amplicons were identified using oligonucleotide microarray probes grouped by virus genus and segment, (A) *Phlebovirus* S segment, (B) *Phlebovirus* M segment, and (C) *Phlebovirus* L segment, and sorted into subgroups based on phylogenetic clade and target virus(es). ECD signals were converted into z-scores. Subgroups with average z-scores greater than 10 were considered positive and used for virus identification. The plotted maximum z-scores represent the greatest individual probe z-score within a subgroup and were used to determine cross-hybridization. The PCR amplicons hybridized with probes in the RVFV subgroups for all three segment targets and *Phlebovirus* generic subgroup for L segment. The virus abbreviations are defined in Table S1.

Microarray identification of phlebovirus GSP PCR amplicons were evaluated using six phleboviruses propagated in cell culture (Table 5). A visual analysis example of RVFV (strain ZH548) is shown in Figure 4. All of the phleboviruses that were tested were PCR positive using the phlebovirus GSPs and 4/6 (67%) were identified to species via all three gene targets. However, sandfly fever Naples virus (SFNV) was not detected by the M segment probes. The five targeted virus species evaluated represents 5/23 (22%) of the total phleboviruses targeted by the ArboChip5.1. The microarray did not contain Chagres virus-specific probes but it was included to evaluate the detection of non-targeted viruses. Chagres virus was detected by the L segment *Phlebovirus* generic probes, showing the detection of non-targeted but related viruses is possible, but virus-specific identification is not.

*Culex pipiens* infected with RVFV strain ZH501 were evaluated alone and in pools of 24 uninfected mosquitoes to simulate field conditions. The microarray identified RVFV via all three gene targets for both preparations.

### Amplification, detection, and identification of orthobunyaviruses

**Figure 5:**
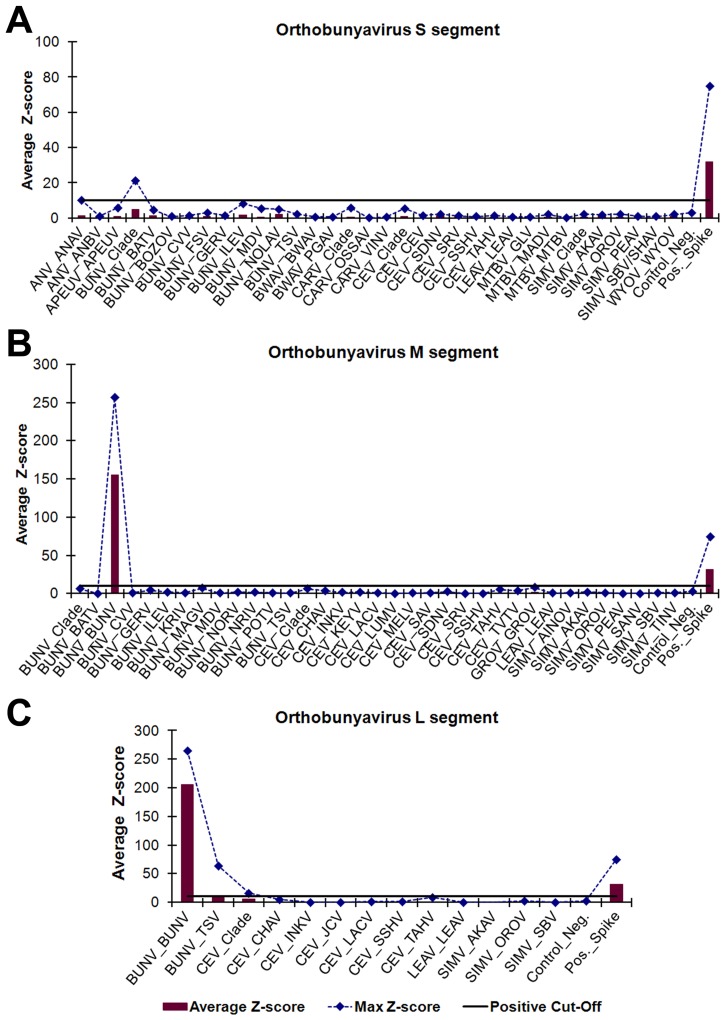
Visual analysis of cell-culture derived BUNV strain 131B-06. *Orthobunyavirus* gene-specific PCR amplicons were identified using oligonucleotide microarray probes grouped by virus genus and segment, (A) *Orthobunyavirus* S segment, (B) *Orthobunyavirus* M segment, and (C) *Orthobunyavirus* L segment, and sorted into subgroups based on phylogenetic clade and target virus(es). ECD signals were converted into z-scores. Subgroups with average z-scores greater than 10 were considered positive and used for virus identification. The plotted maximum z-scores represent the greatest individual probe z-score within a subgroup and were used to determine cross-hybridization. The PCR amplicons hybridized with probes in the BUNV subgroups for the M and L segment targets but virus-specific probes could not be designed for the S segment. The BUNV amplicons did have z-scores greater than 10 for seven of the 42 S segment BUNV clade probes, but the average subgroup z-score was less than 10. The virus abbreviations are defined in Table S1.

Twelve orthobunyaviruses propagated in cell culture were used to evaluate microarray identification of phlebovirus GSP PCR amplicons (Table 6). Example visual analysis of BUNV (strain 131B-06) is shown in Figure 5. The orthobunyaviruses tested could not be PCR amplified using a single pair of S segment GSPs. Therefore two sets of consensus S segments GSPs were created targeting the two major orthobunyavirus groups, the BUNV and California encephalitis virus (CEV) serogroups. Due to a lack of whole genome sequence information publically available and high rates of cross-hybridization of related orthobunyaviruses, Oropouche virus (OROV) and Tahyna virus (TAHV) were the only viruses evaluated that had virus-specific probes designed for all three genome segments. Seven of the 12 (58%) orthobunyaviruses were PCR amplified with three GSP sets. The microarray only detected all three segments from La Crosse virus (LACV); however, the M segment was only identified as belonging to the CEV clade (no M segment specific probes for LACV due to cross-reactivity with other CEV clade viruses). All of the orthobunyavirus tested were PCR amplified, detected, and identified to virus species by at least one target, except for Maguari virus, which was not detected by the microarray due to a lack of PCR amplification of the S and M segments and a lack of virus specific probes for the L segment. Multiple strains of BUNV were used to evaluate microarray identification of virus targets with slight nucleotide differences. The 10 different orthobunyavirus species evaluated represents 10/60 (17%) of the total orthobunyaviruses targeted on the microarray design.

*Ae. aegypti*-infected with LACV was used to evaluate orthobunyavirus detection in mosquitoes. All three segments were PCR amplified and detected by the microarray from single infected mosquitoes.

### Lower limits of detection

Ten-fold serial dilutions of RNA extracted from one infected mosquito pooled with 24 uninfected mosquitoes were used to determine the LLOD for WNVL1 (strain NY397-99), SINV (strain Ken07-611), and RVFV (strain ZH501). Pools of *Cx. pipiens* were used for WNVL1 and RVFV and pools of *Ae. aegypti* were used for SINV. The infected mosquito titers were as follows: WNVL1-infected and RVFV-infected *Cx. pipiens* were 10^4.6^ PFU/mL and 10^6.4^ PFU/mL, respectively, and SINV-infected *Ae. Aegypti* was 10^4.3^ PFU/mL. The LLOD of cDNA by the using the microarray, which includes asymmetric PCR amplification and probe hybridization, was compared to qPCR and conventional PCR. The LLOD data is summarized in Table 7.

**Table 7:** Lower limits of detection from virus-infected mosquito pools.

	Lower limits of detection (PFUe)		
Virus	qPCR	Conv. PCR	Microarray
West Nile	200	200	200
Sindbis	10	10	100
Rift Valley fever	125	125	1,250

The lower limits of detection determined for infected mosquitoes were evaluated by using 10-fold serial dilutions of RNA extracted from one infected mosquito pooled with 24 uninfected mosquitoes. PFUe, plaque forming unit equivalents; qPCR, real-time PCR, Conv. PCR, conventional PCR.

### Evaluation with field-collected mosquitoes

**Figure 6:**
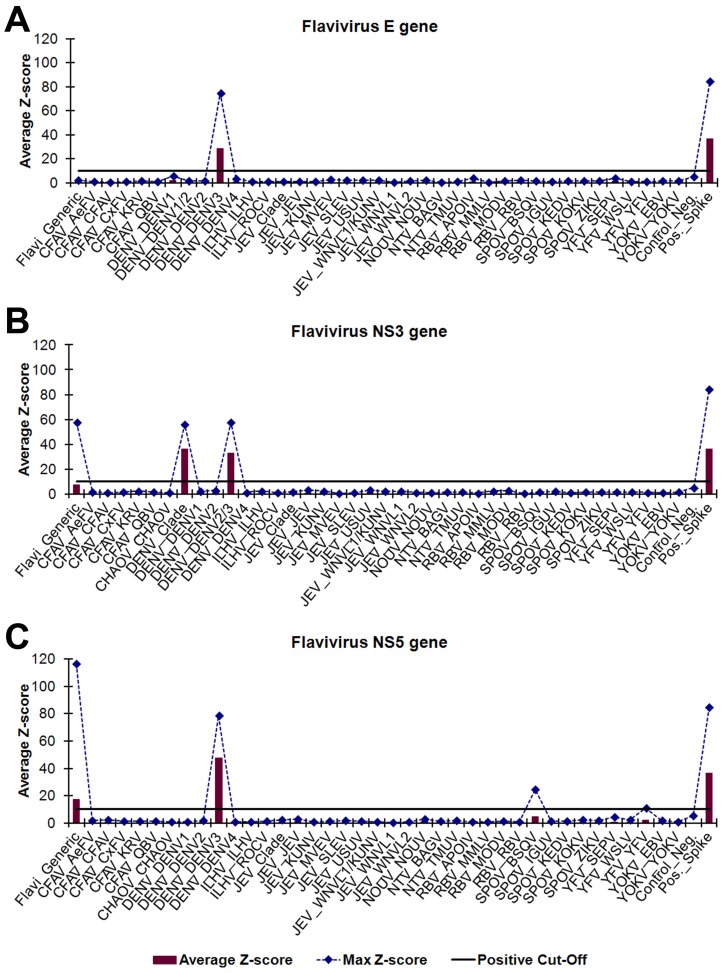
Visual analysis of *Ae. aegypti* mosquito pool Th9-0122 collected in Thailand. Screening methods indicated that the mosquito pool contained flavivirus RNA and cDNA from the pool was PCR amplified with *Flavivirus* (A) E, (B) NS3, and (C) NS5 GSPs. The PCR amplicons hybridized only with probes in the DENV3 subgroups for the (A) E and (C) NS5 gene targets. For the (B) NS3 gene, the amplicons hybridized with probes in the DENV clade and DENV2/3 (hybridizes with DENV2 and DENV3) subgroups, but did not hybridize with the DENV2-specific subgroup. The mosquito pool was identified as containing DENV-3 RNA.

Mosquitoes collected in Thailand were used to determine the applicability of the ArboChip5.1 microarray for identifying virus RNA present in mosquito pools (n = 1–25). From1,445 mosquito pools (642 pools were collected in 2011 and 803 pools were collected in 2012) that were screened for the presence of flaviviruses by conventional PCR, 13 pools were confirmed as containing flavivirus RNA. Selected medically important mosquito pools, such as those containing *Ae. aegypti*, were screened for the presence of alphaviruses, phleboviruses, and BUNV group viruses, however, no positive pools were detected. The flavivirus RNA positive pools were further analyzed using the microarray (Table 8). Visual analysis of the microarray data for sample Th9-0122 is shown in Figure 6 and the raw data analysis are shown in Dataset S1. Nine of the 13 pools were correctly identified by at least one gene targeted by the microarray. RNA sequences related to QBV were found in three pools not identified by the microarray. The other pool not identified by the microarray contained RNA related to Wang Thong virus, a virus not included in the ArboChip5.1 design due to a lack of sequence information in the targeted gene regions. The results were verified by virus isolation and sequencing of the PCR amplicons. Pool number Th9-0032 was identified in the field as QBV, but partial sequencing of the NS5 gene showed that while QBV was its closest match, it was only 78.3% identical. There was less than a 70 bp overlap between the targeted NS5 region that identified Th9-0032 as QBV and the partial sequence. Further analysis on the other genes will need to be completed to determine the identity of the QBV-like virus.

**Table 8:** Microarray identification of flavivirus RNA from Thailand mosquitoes (2011–2012).

		PCR result/microarray identification			
Pool No.^a^	Mosquito species (pool size)	E	NS3	NS5	Sequencing result^b^
Th9-0011	*Cx. quinquefasciatus* (1)	NEG	NEG	POS/CxFV	CxFV
Th9-0024	*Cx. vishnui* (2)	POS/n.d.	NEG	NEG	QBV
Th9-0032	*Cx. quinquefasciatus* (6)	POS/n.d.	POS^c^/n.d.	POS^c^/QBV	QBV-like^d^
Th9-0122	*Ae. aegypti* (1)	POS/DENV3	POS/DENV3	POS/DENV3	DENV3
Th9-0164	*Cx. tritaeniorhynchus* (25)	POS/n.d.	POS/JEV	POS/JEV	JEV
Th9-0175	*Cx. tritaeniorhynchus* (25)	POS/n.d.	POS/JEV	POS/JEV	JEV
Th10-0040	*Cx. vishnui* (20)	NEG	POS/TMUV	POS/n.d.	TMUV
Th10-0121	*Cx. vishnui* (20)	NEG	POS/TMUV	POS/TMUV	TMUV
Th10-0217	*Cx. vishnui* (20)	NEG	POS/TMUV	POS/TMUV	TMUV
Th10-0552	*Cx. vishnui* (20)	NEG	POS/TMUV	POS/TMUV	TMUV
Th10-0578	*Cx. fuscocephala* (16)	NEG	POS/n.d.	NEG	WTV^e^
Th10-0591	*Cx. gelidus* (20)	NEG	NEG	POS^c^/n.d.	QBV
Th10-0767	*Cx. gelidus* (2)	NEG	NEG	POS^c^/n.d.	QBV

cDNAs from mosquito pools that were found to contain viral RNA by flavivirus screening methods were PCR amplified using GSPs and analyzed using the ArboChip5.1 microarray for virus identification. POS, positive PCR amplification; NEG, negative PCR amplification; n.d., no detection; CxFV, *Culex* flavivirus; QBV, Quang Binh virus; DENV3, dengue virus type 3; JEV, Japanese encephalitis virus; TMUV, tembusu virus; WTV, Wang Thong virus.

a Mosquito pool No. beginning with Th9 were collected in 2011, Th10 were collected in 2012.

b Identifcations based on greater than 90% identity using partial NS5 sequences.

c PCR amplification produces a weak visible band.

d Partial NS5 sequence 78.3% identical to QBV.

e WTV targeted probes not included on ArboChip5.1.

Virus-negative pools of *Cx. vishnui*, *Cx. tritaeniorhynchus*, *Cx. quinquefasciatus*, *Cx. gelidus*, *Cx. terzii*, *Ae. albopictus*, *Ae. vexans*, *Mansonia uniformis*, *Armigeres subalbatus*, and *Anopheles peditaeniatus* (n = 19–25 mosquitoes/pool) were processed and used to confirm that the microarray identifications were not from cross-hybridizing mosquito RNA or DNA. All mosquito pools that tested negative by PCR also produced negative microarray results.

## Discussion

The ArboChip5.1 consists of 2,097 oligonucleotide probes targeting 144 different mosquito-borne RNA viruses across the genera *Flavivirus*, *Alphavirus*, *Orthobunyavirus*, and *Phlebovirus*. For most of the selected viruses, capture probes were designed to multiple genes to increase the odds of detection, identification, and confirmation. The assay also utilized consensus GSPs for broad-range PCR amplification of viral RNA, even in the presence of an abundance amount of mosquito nucleic acids. The microarray platform described here, and as we previously discussed, is portable and rugged enough to endure travel and field conditions [30]. The microarray was able to identify viral RNA extracted from cell culture, laboratory-infected mosquitoes, and field-collected mosquitoes, thus making it versatile and practical. To our knowledge, this is the most comprehensive microarray assay specifically designed for arbovirus surveillance in mosquito vectors.

Previously developed and newly defined assays were created or adapted to advance and to streamline the process for using microarrays in field surveillance. RNA extraction and the PCR field-based procedures previously described [39] were modified slightly to fit the needs of the microarray. Asymmetric PCR amplification was utilized to produce a higher ratio of reverse compliment amplicons to hybridize with the microarray probes. In addition, biotinylation of the amplicons was incorporated into the PCR to improve the assay efficiency and ease of use. In a previous study, we evaluated using direct detection of viral RNA without amplification, random primed PCR amplification, and gene-specific PCR amplification and determined that gene-specific PCR amplification was needed for microarray detection of flavivirus RNA in infected mosquitoes [30]. Gene-specific multiplexed PCR was found to reduce the sensitivity of microarray detection (data not shown); therefore, the reactions were performed individually with each GSP set. For a given virus genus, the amplified gene targets were pooled after PCR to reduce the number of microarrays to be tested. Though some level of variability was observed, the outcomes of the microarrays were found to be reproducible. However, the microarray chips have been found to fail on occasion if they were not stored or stripped properly.

The limiting factors for creating the viral diagnostic DNA microarray presented here were the availability of viral sequences present in publically available databases and the sequence diversity in the targeted nucleotide regions. Flaviviruses and alphaviruses are generally well characterized, and their entire genomic sequences are available for most known species. As a result, unique probes targeting all three genes of diagnostic interest for most flaviviruses and alphaviruses were created. We were able to detect and identify all of the flaviviruses and alphaviruses tested from both cell culture and infected mosquitoes, except for a few closely related alphaviruses as discussed below. Closely related flaviviruses, such as the four serotypes of DENV and WNVL1, WNVL2, and KUNV were clearly differentiated using the microarray. Additionally, JEV and WNVL1, both JEV clade flaviviruses, were detected from a pool of laboratory infected mosquitoes, showcasing the ability of the microarray to differentiate between closely related viruses that might be found in the same mosquito pool. Though we were able to differentiate between many closely related alphaviruses, we were not able to differentiate between SINV, OCKV, and BBKV and between ONNV and IOV. This was because there was not enough sequence diversity between the listed alphaviruses in the targeted nucleotide regions to create probes that would not cross-hybridize using the described assay conditions. Instead, the viruses were identified as either part of the SINV or ONNV clades and other data, such as the mosquito species and collection location, could be used to distinguish the specific alphavirus.

Some bunyaviruses, such as BUNV (*Orthobunyavirus*), LACV (*Orthobunyavirus*), and RVFV (*Phlebovirus*), are very well studied and genetically characterized. However, the *Bunyaviridae* family is expansive and diverse, and despite many characterization efforts [43], [45], [46], [47], [48], many bunyavirus genomes have not been fully sequenced. Sequence information is especially lacking for the L segment for many of the orthobunyaviruses, as evident by our nominal L segment probes and was the reason why we could not design probes to all three segments for most of the evaluated orthobunyaviruses. Even in some cases where complete sequence information was available, virus-specific probes could not be designed for all of the segments due to sequence similarities. For instance, the S segment of orthobunyaviruses share close sequence identity [45], making microarray differentiation of this segment difficult. Moreover, bunyaviruses, like many other segmented viruses, can use natural genome reassortment as a driving force for evolution [49], [50]. For example, Ngari virus is the reassortment of two other orthobunyaviruses: the S and L segments from BUNV and the M segment from Batai virus [51], [52]. These factors combined made it difficult to design virus-specific probes to all three segments for many of these viruses. However, there was enough sequence data available and nucleotide diversity to design probes to make microarray identification of at least one segment for the orthobunyaviruses and phleboviruses tested.

Field-collected mosquitoes have varied arbovirus titers based on the virus strain, mosquito species, environmental conditions, and infection status (nondisseminated limited to the midgut or disseminated throughout the mosquito's body) [53], [54]. Dilutions of mosquito pools containing a single mosquito with a known disseminated infection with WNV, SINV, or RVFV and 24 uninfected mosquitoes were made to mimic the virus titer variations. The LLODs were determined for viral RNA present in pools of laboratory infected mosquitoes and were compared to modified qPCR and conventional PCR assays. The microarray assay was expected to be less sensitive than the PCR assays because the microarray has an additional detection bottleneck at the point of probe hybridization. Yet the microarray LLODs compared to the PCR assays were at most only a log greater, making it suitable by comparison for diagnostic use. If greater sensitivities are desired, longer hybridization times (data not shown) and post-hybridization signal amplification [37] would help increase detection of low virus titers. However, the methods described here were optimized to reduce the assay time while maintaining a sensitivity level adequate for analyzing field-collected samples. This was evaluated in Thailand in the springs of 2011 and 2012 using various numbers of pooled mosquitoes. From the field collections, RNA from medically relevant viruses, including DENV-3 and JEV, and TMUV, a virus known to cause severe disease in ducks [55], was identified. Viral RNA from two arthropod-specific flaviviruses, CxFV and a QBV-like virus, were also identified, though RNA isolated from three other flavivirus PCR positive pools could not be identified. After passage of the PCR positive samples in cultured cells, QBV RNA was identified using the microarray, suggesting that the level of QBV RNA in the mosquito pools was below the microarray's LLOD. Bolling *et al.* noticed a bimodal distribution of CxFV in a naturally infected *Cx. pipiens* laboratory colony [56] which, along with the other factors mentioned above for varied titers, may explain why some arthropod-specific flaviviruses from field samples were below microarray's LLOD.

The intended use of the ArboChip5.1 is for arbovirus surveillance studies targeting mosquitoes. The concept is to use generic screening assays to identify mosquito pools potentially containing arboviral RNA. The screening assays should be based on the field or laboratory team's capabilities and can include the conventional and real-time assays cited in this paper or other published assays [36], [40], [41], [42], [43], [44], [57], [58]. Viral RNA positive mosquito pools would then be PCR amplified using the GSPs for the genus (or genera) of interest and analyzed using the microarray platform. These PCR assays should not be used alone for screening mosquito pools for arbovirus RNA because 1) asymmetric PCR amplification can reduce sensitivity, 2) biotin would be wasted on many negative pools, increasing the costs of surveillance and 3) the degenerate GSP sets were designed to increase assay sensitivity, but at the cost of specificity, meaning additional non-specific bands are produced which can confound interpretation of the results. The microarray assay could be used to analyze viral RNA isolated from animal tissue or serum, or from sand flies, because many phleboviruses are included in the design. However, viral RNA isolated from these sources has not been evaluated using the microarray and associated methods.

Multiplexed-PCR methods for viral RNA identification can be efficient for a small sub-set of viruses, yet when there is a need to discern between a larger set of mosquito-borne viruses, PCR approaches for virus identification can become laborious. The microarray, as described here, is a time and cost effective method for the detection of numerous viral RNA species potentially present within an infected mosquito with limited bias. Each ElectraSense 4×2K microarray has four wells to analyze four samples at a time, and when using the rotisserie hybridization incubator described here, holding four microarray chips at a time, up to 16 samples can be processed in as little as 12 hours. The major limitations of this assay are that only known viral sequences can be used for probe design, meaning that it cannot identify novel viruses, and it cannot discern between slight nucleotide changes, meaning that it cannot anticipate viral evolution. Even though previously unknown or non-targeted viral RNA maybe detected because of some sequence homology in one of the targeted regions, the microarray would not be able to identify novel viruses. On the other hand, next-generation sequencing (NGS) has the potential to provide the most amount of nucleic acid information, but they are not portable and require more time and labor to create sequencing libraries and analyze the results. Moreover, NGS systems are much more expensive to operate, generally costing over $1000 per run [59]. To compare, ElectraSense 4×2K microarray chips costs approximately $500 each and can be used four to five times, thus costing $25 to $31.25 per sample.

In summary, the ArboChip5.1 microarray can identify multiple genes from a wide range of mosquito-borne RNA viruses through broad-range PCR amplification and detection with virus-specific probes. It is vastly more multiplexed than PCR assays alone, more specific than universal microarrays, and more cost effective than most sequencing platforms. The microarray reader is small and rugged, making it field-portable. This system is an ideal tool for active surveillance or monitoring programs in regions where little is known about the circulating mosquito-borne viruses. We have demonstrated that it detects many viruses of medical importance, such as DENV, YFV, JEV, and CHIKV. In addition, the microarray targets many viruses that are also major causes of animal disease, such as RVFV, VEEV, and WNV. The ultimate goal is to provide researchers, veterinarians, and clinicians with a diagnostic tool that will allow them to recognize previously known pathogens that are emerging or re-emerging before they become major health issues.

## Supporting Information

Dataset S1 **Example data analysis.** Data analysis worksheet with example data from the field collected mosquito pool Th9-0122 (Figure 6, Table 8). Individual ECD signals and calculated z-scores are shown for each probe. The data summary shows the average z-scores and maximum z-scores from each subgroup. Visual analysis of the subgroups is provided for each group (virus genus and gene target).
(XLSX)

Figure S1 **Detection of JEV and WNVL1 from a dual-infected pool of** ***Cx. pipiens*** **mosquitoes.** A mosquito pool (n = 25) containing two infected *Cx. pipiens*, one each with JEV and WNVL1, and 23 uninfected mosquitoes were processed together and PCR amplified using the *Flavivirus* GSP sets. The NS3 PCR amplicons hybridized with probes in the JEV-specific, WNVL1/KUNV, and WNVL1-specific subgroups. The JEV and WNVL1 probes have not been observed to cross-hybridize to the other virus when analyzed individually, thus showing the detection of RNA from two related but distinct flaviviruses in a single mosquito pool. The virus abbreviations are defined in Table S1.
(TIF)

Table S1 **Target viruses.** List of the ArboChip5.1 targeted mosquito-borne RNA viruses, sorted by genera and clades (as determined by phylogenetic comparisons of the gene target sequences and not necessarily represented by taxonomic clades), with the number of virus-specific and non-specific probes.
(XLSX)

Table S2 **Probe list.** List of the ArboChip5.1 oligonucleotide probes created for the multi-gene detection of RNA from mosquito-borne viruses. For each of 2,097 probes, the probe name, group (genus and gene target), subgroup (clade and virus), sequence source (GenBank accession number), start position, design source, and sequence is listed.
(XLSX)

## References

[1] Weaver SC, Reisen WK (2010) Present and future arboviral threats. Antiviral Res 82: 328. 10.1016/j.antiviral.2009.10.008 PMC2815176 19857523

[2] Lindenbach BD, Rice CM (2003) Molecular biology of flaviviruses. Adv Virus Res 59: 23–61. 14696326 10.1016/s0065-3527(03)59002-9

[3] Stollar V, Thomas VL (1975) An agent in the *Aedes aegypti* cell line (Peleg) which causes fusion of *Aedes albopictus* cells. Virology 64: 367–377. 806166 10.1016/0042-6822(75)90113-0

[4] Crabtree MB, Sang RC, Stollar V, Dunster LM, Miller BR (2003) Genetic and phemotypic characterization of the newly described insect flavivirus, Kamiti River virus. Arch Virol 148: 1095–118. 12756617 10.1007/s00705-003-0019-7

[5] Sang RC, Gichogo A, Gachoya J, Dunster MD, Ofula V, et al (2003) Isolation of a new flavivirus related to cell fusing agent virus (CFAV) from field-collected flood-water *Aedes* mosquitoes sampled from a dambo in central Kenya. Arch Virol 148: 1085–1093. 12756616 10.1007/s00705-003-0018-8

[6] Cook S, Bennet SN, Holmes EC, De Chesse R, Moureau G, et al (2006) Isolation of a new strain of the flavivirus cell fusing agent virus in a natural mosquito population from Puerto Rico. J Gen Virol 87: 735–748. 16528021 10.1099/vir.0.81475-0

[7] Hoshino K, Isawa H, Tsuda Y, Yano K, Sasaki T, et al (2007) Genetic characterization of a new insect flavivirus isolated from *Culex pipiens* mosquito in Japan. Virology 359: 405–414. 17070886 10.1016/j.virol.2006.09.039

[8] Crabtree MB, Nga PT, Miller BR (2009) Isolation and characterization of a new mosquito flavivirus, Quang Binh virus, from Vietnam. Arch Virol 154: 857–860. 19347244 10.1007/s00705-009-0373-1

[9] Bolling BG, Eisen L, Moore CG, Blair CD (2011) Insect-specific flaviviruses from Culex mosquitoes in Colorado, with evidence of vertical transmission. Am J Trop Med Hyg 85: 169–177. 21734144 10.4269/ajtmh.2011.10-0474 PMC3122363

[10] Strauss JH, Strauss EG (1994) The alphaviruses: gene expression, replication, and evolution. Microbiol Rev 58: 491–562. 7968923 10.1128/mr.58.3.491-562.1994 PMC372977

[11] Elliott RM, Bouloy M, Calisher CH, Goldbach R, Moyer JT, et al.. (2000) Family Bunyaviridae. In: van Regenmortel MHV, Fauguet CM, Bishop DHL editors. Virus Taxonomy: Seventh Report of the International Committee on Taxonomy of Viruses. San Diego: Academic Press. pp. 599–621.

[12] Meegan JM, Bailey CL (1988) Rift Valley fever. In: Monath TP editor. The Arboviruses: Epidemiology and Ecology, Vol. IV. Boca Raton, FL: CRC Press. pp. 61–76.

[13] Jones KE, Patel NG, Levy MA, Storeygard A, Balk D, et al (2008) Global trends in emerging infectious diseases. Nature 451: 990–994. 18288193 10.1038/nature06536 PMC5960580

[14] Hollidge BS, González-Scarano F, Soldan SS (2010) Arboviral encephalitides: transmission, emergence, and pathogenesis. J Neuroimmune Pharmacol 5: 428–442. 20652430 10.1007/s11481-010-9234-7 PMC3286874

[15] Meegan JM (1979) Rift Valley fever in Egypt: an overview of the epizootics in 1977 and 1978. Contrib Epidemiol Biostat 3: 100–113.

[16] Nguku PM, Sharif SK, Mutonga D, Amwayi S, Omolo J, et al (2010) An investigation of a major outbreak of Rift Valley fever in Kenya: 2006–2007. Am J Trop Med Hyg 88: 5–13. 10.4269/ajtmh.2010.09-0288 PMC2913496 20682900

[17] Briese T, Jia XY, Huang C, Grady LJ, Lipkin WI (1999) Identification of a Kunjin/West Nile-like flavivirus in brains of patients with New York encephalitis. Lancet 354: 1650. 10.1016/s0140-6736(99)04576-6 10520637

[18] Brinton MA (2002) The molecular biology of West Nile virus: a new invader of the western hemisphere. Annu Rev Microbiol 56: 371–402. 12142476 10.1146/annurev.micro.56.012302.160654

[19] Kariuki Njenga M, Nderitu L, Ledermann JP, Ndirangu A, Logue CH, et al (2008) Tracking epidemic Chikungunya virus into the Indian Ocean from East Africa. J Gen Virol 89: 2754–2760. 18931072 10.1099/vir.0.2008/005413-0 PMC3347796

[20] CDC Guidelines for Arbovirus Surveillance in the US, CDC Division of Vector-Borne Infectious Diseases (DVBID). http://www.cdc.gov/ncidod/dvbid/Arbor/arboguid.htm (Accessed 05 January 2013).

[21] Wang D, Coscoy L, Zylberberg M, Avila PC, Boushey HA, et al (2002) Microarray-based detection and genotyping of viral pathogens. PNAS 99: 15687–15692. 12429852 10.1073/pnas.242579699 PMC137777

[22] Wang D, Urisman A, Liu YT, Springer M, Ksiazek TG, et al (2003) Viral discovery an sequence recovery using DNA microarrays. PLoS Bio 1: 257–260. 10.1371/journal.pbio.0000002 PMC261870 14624234

[23] Palacios G, Quan PL, Hirschberg DL, Liu Y, Zhai J, et al (2007) Panmicrobial oligonucleotide array for diagnosis of infectious diseases. EID 13: 73–81. 10.3201/eid1301.060837 PMC2725825 17370518

[24] Korimbocus J, Scaramozzino N, Lacroix B, Crance JM, Garin D, et al (2005) DNA probe array for the simultaneous identification of herpesviruses, enteroviruses, and flaviviruses. J Clin Microbiol 43: 3779–3787. 16081910 10.1128/JCM.43.8.3779-3787.2005 PMC1233982

[25] Nordstrom H, Falk KI, Lindegren G, Mouzavi-Jazi M, Walden A, et al (2005) DNA microarray technique for detection and identification of seven flaviviruses pathogenic for man. J Med Virol 77: 528–540. 16254977 10.1002/jmv.20489

[26] Chou C, Lee T, Chen C, Hsiao H, Lin Y, et al (2006) Design of microarray probes for virus identification and detection of emerging viruses at the genus level. BMC Bioinfo 7: 232. 10.1186/1471-2105-7-232 PMC1523220 16643672

[27] Putonti C, Chumakov S, Mitra R, Fox GE, Wilson RC, et al (2006) Human-blind probes and primers for dengue virus identification. FEBS J 273: 398–408. 16403026 10.1111/j.1742-4658.2005.05074.x

[28] Xiao-Ping K, Yong-Qiang L, Qing-Ge S, Hong L, Quing-Yu Z, et al (2009) Development of a consensus microarray method for identification of some highly pathogenic viruses. J Med Virol 81: 1945–1950. 19774692 10.1002/jmv.21602 PMC7166427

[29] Berthet N, Paulous S, Coffey LL, Frenkiel MP, Moltini I, et al (2012) Resequencing microarray method for molecular diagnosis of human arboviral diseases. J Clin Virol 56: 322–327. 10.1016/j.jcv.2012.10.022 23219893

[30] Grubaugh ND, Petz LN, Melanson VR, McMenamy SS, Turell MJ, et al (2012) Evaluation of a field-portable DNA microarray platform and nucleic acid amplification strategies for the detection of arboviruses, arthropods, and bloodmeals. Am J Trop Med Hyg [Epub ahead of print]. 10.4269/ajtmh.2012.12-0048 PMC3583313 23249687

[31] Roth KM, Peyvan K, Schwarzkopf KR, Ghindilis AL (2006) Elecetrochemical detection of short DNA oligomer hybridization using the CombiMatrix microarray reader. Electroanalysis 18: 1982–1988.

[32] Ghindilis AL, Smith MW, Schwarzkopf KR, Roth KM, Peyvan K, et al (2007) CombiMatrix oligonucleotide arrays: genotyping and gene expression assays employing electrochemical detection. Biosens Bioelectron 22: 1853–1860. 16891109 10.1016/j.bios.2006.06.024

[33] Lodes MJ, Suciu D, Elliot M, Stover AG, Ross M, et al (2006) Use of a semiconductor-based oligonucleotide microarray for Influenza A virus subtype identification and sequencing. J Clin Microbiol 44: 1209–1218. 16597840 10.1128/JCM.44.4.1209-1218.2006 PMC1448669

[34] Lodes MJ, Suciu D, Willmoth JL, Ross M, Munro S, et al (2007) Identification of upper respiratory tract pathogens using electrochemical detection on an oligonucleotide microarray. PLoS One 2: e924. 17895966 10.1371/journal.pone.0000924 PMC1976596

[35] Maher-Sturgess SL, Forrester NL, Wayper PJ, Gould EA, Hall RA, et al (2008) Universal primers that amplify RNA from all three flavivirus subgroups. Virol J 5: 16. 18218114 10.1186/1743-422X-5-16 PMC2263041

[36] Eshoo MW, Whitehouse CA, Zoll ST, Massire C, Pennella TD, et al (2007) Direct broad-range detection of alphaviruses in mosquito extracts. Virology 368: 286–295. 17655905 10.1016/j.virol.2007.06.016

[37] Wojciechowski J, Chase-Baldwin K, Wasieloski LP Jr, Padilla S, Vora GJ, et al (2010) Enhancement of deoxyribonucleic acid microarray performance using post-hybridization signal amplification. Anal. Chim. Acta 679: 85–90. 10.1016/j.aca.2010.09.007 20951861

[38] Rosen L, Gubler D (1974) The use of mosquitoes to detect and propagate dengue viruses. Am J Trop Med Hyg 23: 1153–1160. 4429185 10.4269/ajtmh.1974.23.1153

[39] O'Guinn ML, Lee JS, Kondig JP, Fernandez R, Carbajal F (2004) Field detection of Eastern equine encephalitis virus in the Amazon Basin region of Peru using reverse transcription-polymerase chain reaction adapted for field identification of arthropod-borne pathogens. Am J Trop Med Hyg 70: 164–171. 14993628

[40] Chao D, Davis BS, Chang GJ (2007) Development of multiplex real-time reverse transcriptase PCR assays for detecting eight medically important flaviviruses in mosquitoes. J Clin Micro 45: 584–589. 10.1128/JCM.00842-06 PMC1829073 17108075

[41] Drosten C, Göttig S, Schilling S, Asper M, Panning M, et al (2002) Rapid detection and quantification of RNA of Ebola and Marbug viruses, Lassa virus, Crimean-Congo hemorrhagic fever virus, Rift Valley fever virus, dengue virus, and yellow fever virus by real-time reverse transcription-PCR. J Clin Microbiol 40: 2323–2330. 12089242 10.1128/JCM.40.7.2323-2330.2002 PMC120575

[42] Kuno G (1998) Universal diagnostic RT-PCR protocol for arboviruses. J Virol Meth 72: 27–41. 10.1016/s0166-0934(98)00003-2 9672130

[43] Lambert AJ, Lanciotti RS (2009) Consensus amplification and novel multiplex sequencing method for S segment species identification of 47 viruses of the Orthobunyavirus, Phlebovirus, and Nairovirus genera of the family Bunyaviridae. J Clin Micro 47: 2398–2404. 10.1128/JCM.00182-09 PMC2725646 19535518

[44] Kuno G, Mitchel C, Chang GJ, Smith G (1996) Detecting bunyaviruses of the Bunyamwera and California serogroups by a PCR technique. J Clin Micro 34: 1184–1188. 10.1128/jcm.34.5.1184-1188.1996 PMC228979 8727900

[45] Lambert AJ, Lanciotti RS (2008) Molecular characterization of medically important viruses of the genus Orthobunyavirus. J Gen Virol 89: 2580–2585. 18796727 10.1099/vir.0.2008/002253-0

[46] Mores CN, Turell MJ, Dyer J, Rossi CA (2009) Phylogenetic relationships among orthobunyaviruses isolated from mosquitoes captured in Peru. Vector Borne Zoonotic Dis 9: 25–32. 18759638 10.1089/vbz.2008.0030

[47] Chowdhary R, Street C, Travassos da Rossa A, Nunes MR, Tee KK, et al (2012) Genetic characterization of the Wyeomyia group of orthobunyaviruses and their phylogenetic relationships. J Gen Virol 93: 1023–1034. 22278828 10.1099/vir.0.039479-0 PMC3541803

[48] Hang J, Forshey BM, Kochel TJ, Li T, Solórzano VF, et al (2012) Random amplification and pyrosequencing for identification of novel viral genome sequences. J Biomol Tech 23: 4–10. 22468136 10.7171/jbt.12-2301-001 PMC3313696

[49] Turell MJ, Saluzzo J-F, Tammariello RF, Smith JF (1990) Generation and transmission of Rift Valley fever viral reassortants by the mosquito *Culex pipiens* . J Gen Virol 71: 2307–2312. 2230736 10.1099/0022-1317-71-10-2307

[50] Briese T, Kapoor V, Lipkin WI (2007) Natural M-segment reassortment in Potosi and Main Drain viruses: implications for the evolution of orthobunyaviruses. Arch Virol 152: 2237–2247. 17891328 10.1007/s00705-007-1069-z

[51] Briese T, Bird B, Kapoor V, Nichol ST, Lipkin WI (2006) Batai and Ngari viruses: M segment reassortment and association with severe febrile disease outbreaks in East Africia. J Virol 80: 5627–5630. 16699043 10.1128/JVI.02448-05 PMC1472162

[52] Yanase T, Kato T, Yamakawa M, Takayoshi K, Nakamura K, et al (2006) Genetic characterization of Batai virus indicates a genomic reassortment between orthobunyaviruses in nature. Arch Virol 151: 2253–2260. 16820982 10.1007/s00705-006-0808-x

[53] Nasci RS, Mitchell CJ (1996) Arbovirus titer variation in field-collected mosquitoes. J Am Mosq Control Assoc 12: 167–171. 8827588

[54] Turell MJ, Gargan TPII, Bailey CL (1984) Dissemination and replication of Rift Valley fever virus in *Culex pipiens* . Am J Trop Med Hyg 33: 176–181. 6696176 10.4269/ajtmh.1984.33.176

[55] Tang Y, Diao Y, Gao X, Yu C, Chen L, et al (2012) Analysis of the complete genome of Tembusu virus, a flavivirus isolate from ducks in China. Transbound Emerg Dis 59: 336–343. 22103739 10.1111/j.1865-1682.2011.01275.x

[56] Bolling BG, Olea-Popelka FJ, Eisen L, Moore CG, Blair CD (2012) Transmission dynamics of an insect-specific flavivirus in a naturally infected *Culex pipiens* laboratory colony and effects of co-infection on vector competence for West Nile virus. Virology 427: 90–97. 22425062 10.1016/j.virol.2012.02.016 PMC3329802

[57] Grywna K, Kupfer B, Panning M, Drexler JF, Emmerich P, et al (2010) Detection of all species of the genus Alphavirus by reverse transcription-PCR with diagnostic sensitivity. J Clin Microbiol 48: 2286–3387. 10.1128/JCM.00317-10 PMC2937745 20504990

[58] Yang CF, Chen CF, Su CL, Teng HJ, Lu LC, et al (2010) Screening of mosquitoes using SYBR Green I-based real-time RT-PCR with group-specific primers for detection of Flaviviruses and Alphaviruses in Taiwan. J Virol Methods 168: 147–151. 20471427 10.1016/j.jviromet.2010.05.006

[59] Liu L, Li Y, Hu N, He Y, Pong R, et al (2012) Comparison of next-generation sequencing systems. J Biomed Biotechnol 2012: 251364. 22829749 10.1155/2012/251364 PMC3398667

