# Supplementary figures and images for: Multi-Gene Detection and Identification of Mosquito-Borne RNA Viruses Using an Oligonucleotide Microarray
Source: PLoS Negl Trop Dis. 2013 Aug 15;7(8):e2349. doi: 10.1371/journal.pntd.0002349 (PMC3744434; doi:10.1371/journal.pntd.0002349)

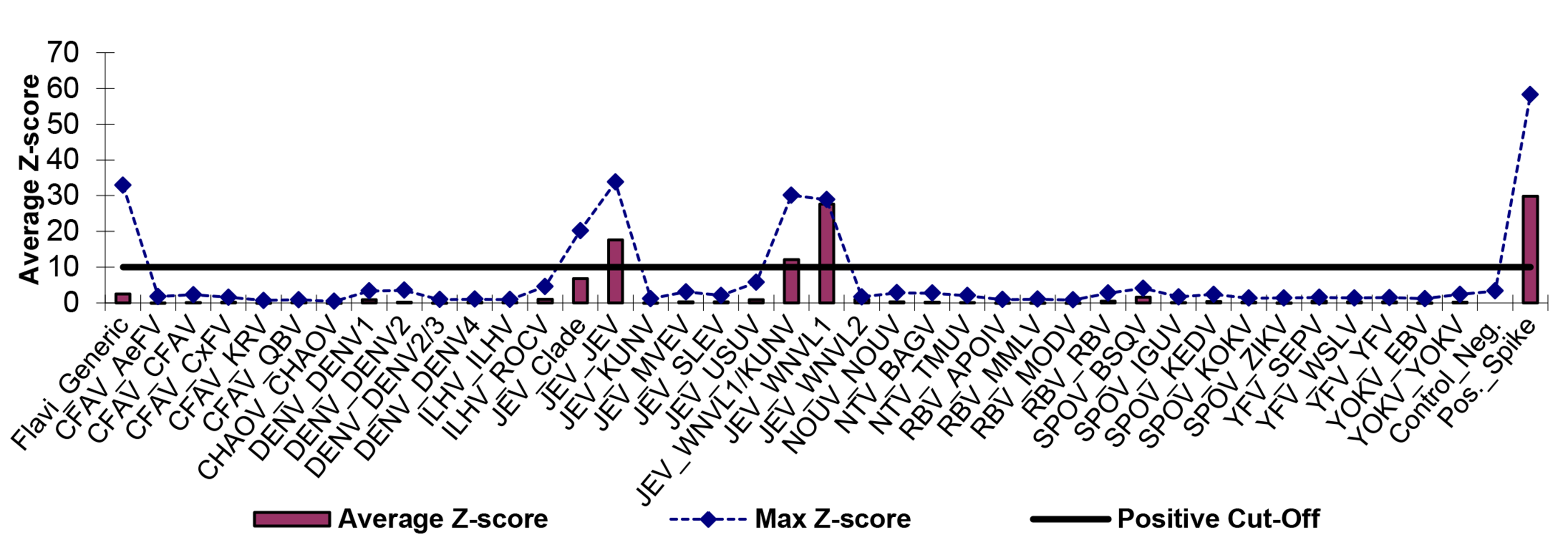

Supplement: Figure S1 — Detection of JEV and WNVL1 from a dual-infected pool of Cx. pipiens mosquitoes. A mosquito pool (n = 25) containing two infected Cx. pipiens, one each with JEV and WNVL1, and 23 uninfected mosquitoes were processed together and PCR amplified using the Flavivirus GSP sets. The NS3 PCR amplicons hybridized with probes in the JEV-specific, WNVL1/KUNV, and WNVL1-specific subgroups. The JEV and WNVL1 probes have not been observed to cross-hybridize to the other virus when analyzed individually, thus showing the detection of RNA from two related but distinct flaviviruses in a single mosquito pool. The virus abbreviations are defined in Table S1. (TIF) [file pntd.0002349.s002.tif]
